# [μ-1,1′-Bis(diphenyl­phosphino)ferrocene-κ^2^ *P*:*P*′]bis­{[(*Z*)-*O*-isopropyl-*N*-(4-methyl­phen­yl)thio­carbamato-κ*S*]gold(I)}

**DOI:** 10.1107/S1600536809047898

**Published:** 2009-11-18

**Authors:** Primjira P. Tadbuppa, Edward R. T. Tiekink

**Affiliations:** a Department of Chemistry, National University of Singapore, Singapore 117543; b Department of Chemistry, University of Malaya, 50603 Kuala Lumpur, Malaysia

## Abstract

In the title compound, [Au_2_Fe(C_11_H_14_NOS)_2_(C_17_H_14_P)_2_], the Fe^II^ atom is located on a crystallographic centre of inversion. For the Au^I^ atom, the deviation from linearity defined by its *S*,*P*-donor set [S—Au—P = 178.17 (8) Å] is due to an intra­molecular Au⋯O contact [3.079 (4) Å]. In the crystal, supra­molecular chains mediated by C—H⋯N inter­actions are formed, which run parallel to [001].

## Related literature

For structural systematics and luminescence properties of phosphinegold(I) carbonimidothio­ates, see: Ho *et al.* (2006 ▶); Ho & Tiekink (2007 ▶); Kuan *et al.* (2008 ▶). For the synthesis, see Hall *et al.* (1993 ▶). For a related structure, see Ho & Tiekink (2009 ▶).
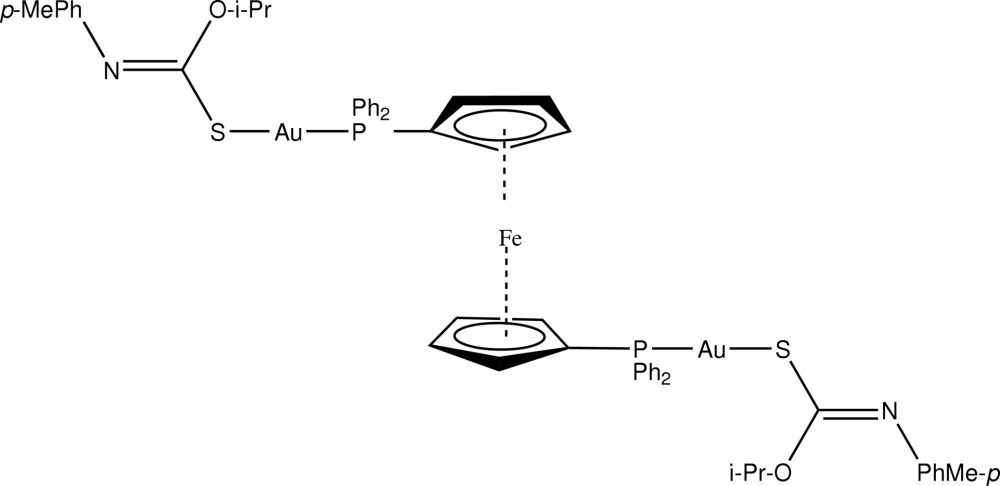


## Experimental

### 

#### Crystal data

[Au_2_Fe(C_11_H_14_NOS)_2_(C_17_H_14_P)_2_]
*M* *_r_* = 1364.87
Triclinic, 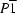

*a* = 8.1631 (9) Å
*b* = 13.4959 (14) Å
*c* = 13.5154 (14) Å
α = 107.440 (2)°
β = 97.401 (2)°
γ = 106.366 (2)°
*V* = 1325.5 (2) Å^3^
*Z* = 1
Mo *K*α radiation
μ = 5.97 mm^−1^
*T* = 223 K
0.21 × 0.07 × 0.04 mm

#### Data collection

Bruker SMART CCD diffractometer
Absorption correction: multi-scan (*SADABS*; Bruker, 2000 ▶) *T*_min_ = 0.629, *T*_max_ = 1
7710 measured reflections
4651 independent reflections
3790 reflections with *I* > 2σ(*I*)
*R*_int_ = 0.036

#### Refinement

*R*[*F* ^2^ > 2σ(*F* ^2^)] = 0.040
*wR*(*F* ^2^) = 0.088
*S* = 0.98
4651 reflections
305 parameters
H-atom parameters constrained
Δρ_max_ = 1.74 e Å^−3^
Δρ_min_ = −0.93 e Å^−3^

### 

Data collection: *SMART* (Bruker, 2000 ▶); cell refinement: *SAINT* (Bruker, 2000 ▶); data reduction: *SAINT*; program(s) used to solve structure: *PATTY* in *DIRDIF92* (Beurskens *et al.*, 1992 ▶); program(s) used to refine structure: *SHELXL97* (Sheldrick, 2008 ▶); molecular graphics: *DIAMOND* (Brandenburg, 2006 ▶); software used to prepare material for publication: *publCIF* (Westrip, 2009 ▶).

## Supplementary Material

Crystal structure: contains datablocks global, I. DOI: 10.1107/S1600536809047898/hb5221sup1.cif

Structure factors: contains datablocks I. DOI: 10.1107/S1600536809047898/hb5221Isup2.hkl

Additional supplementary materials:  crystallographic information; 3D view; checkCIF report

## Figures and Tables

**Table 1:** Selected bond lengths (Å)

Au—S1	2.2883 (19)
Au—P1	2.2520 (17)

**Table 2:** Hydrogen-bond geometry (Å, °)

*D*—H⋯*A*	*D*—H	H⋯*A*	*D*⋯*A*	*D*—H⋯*A*
C25—H25⋯N1^i^	0.94	2.45	3.370 (11)	167

Symmetry code: (i) 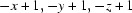.

**Fig. 1.**
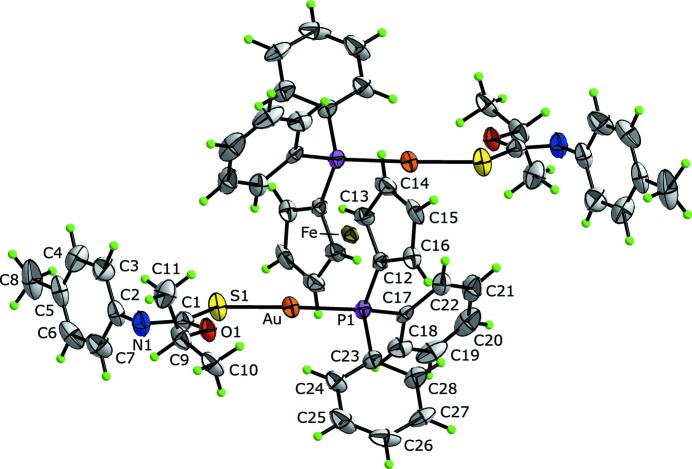
Molecular structure of the dinuclear complex (I) showing displacement ellipsoids at the 50% probability level. The molecule is located about a centre of inversion and unlabelled atoms are related by the symmetry operation 1 - x, 1 - y, -z.

**Fig. 2.**
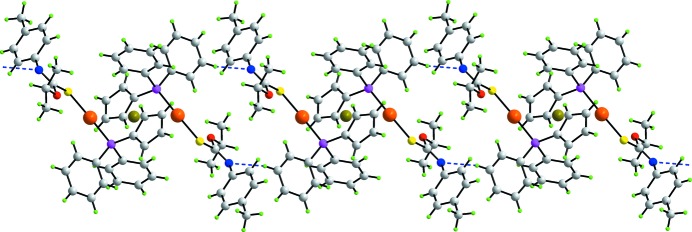
Supramolecular chain in (I) mediated by C—H···N contacts (blue dashed lines). Colour code: Au, orange; S, yellow; P, pink; O, red; N, blue; C, grey; and H, green.

## supplementary crystallographic information

### Comment

As a part of systematic studies of phosphinegold(I) thiocarbamides (Ho *et al.* 2006; Ho & Tiekink, 2007; Kuan *et al.*, 2008), the title compound, dppf{Au[SC(O-^i^Pr)NC_6_H_4_Me-p]}_2_, was synthesized, (I); dppf is (Ph_2_PC_5_H_4_)_2_Fe. The dinuclear molecule has crystallographic symmetry with the Fe atom lying on an inversion centre, Fig. 1.

The gold atom exists in the expected linear geometry defined by a SP donor set, Table 1, and the deviation from linearity [S1–Au–P1 is 178.17 (8) Å] is ascribed to the close approach of the O1 atom, Au···O = 3.079 (4) Å. The anion, with a *Z* configuration about the C1═N1 bond, shows the expected characteristics. The magnitudes of C1—S1 and C1═N1 of 1.753 (7) and 1.253 (9) Å, respectively, confirm that the anion is coordinating as a thiolate. The overall conformation of the molecule is "open" in that the thiocarbamate ligands are lying on either side of the molecule. This contrasts the situation in each of dppf{Au[SC(OR)NC_6_H_4_NO_2_-p]}_2_, for *R* = Me (Ho *et al.*, 2006) and ^i^Pr (Ho & Tiekink, 2009), whereby the molecule has a U-shaped conformation allowing for the formation of intramolecular Au···Au interactions.

In the crystal structure of (I), supramolecular chains are formed down the *c* axis owing to the presence of C–H···N interactions, Table 2 and Fig. 2.

### Experimental

Compound (I) was prepared following the standard literature procedure from the reaction of dppf(AuCl)_2_ and (^i^Pr)OC(S)N(H)C_6_H_4_Me-p in the presence of base (Hall *et al.*, 1993).

### Refinement

The H atoms were geometrically placed (C—H = 0.94–0.99 Å) and refined as riding with *U*_iso_(H) = 1.2–1.5*U*_eq_(C). The maximum and minimum residual electron density peaks of 1.74 and 0.93 e Å^-3^, respectively, were located 1.03 Å and 1.02 Å from the Au atom.

### Crystal data

**Table:**

[Au_2_Fe(C_11_H_14_NOS)_2_(C_17_H_14_P)_2_]	*Z* = 1
*M**_r_* = 1364.87	*F*(000) = 668
Triclinic, *P*1	*D*_x_ = 1.710 Mg m^−^^3^
Hall symbol: -P 1	Mo *K*α radiation, λ = 0.71069 Å
*a* = 8.1631 (9) Å	Cell parameters from 2346 reflections
*b* = 13.4959 (14) Å	θ = 2.7–30.0°
*c* = 13.5154 (14) Å	µ = 5.97 mm^−^^1^
α = 107.440 (2)°	*T* = 223 K
β = 97.401 (2)°	Block, orange
γ = 106.366 (2)°	0.21 × 0.07 × 0.04 mm
*V* = 1325.5 (2) Å^3^	

### Data collection

**Table:**

Bruker SMART CCD diffractometer	4651 independent reflections
Radiation source: fine-focus sealed tube	3790 reflections with *I* > 2σ(*I*)
graphite	*R*_int_ = 0.036
ω scans	θ_max_ = 25.0°, θ_min_ = 1.6°
Absorption correction: multi-scan (*SADABS*; Bruker, 2000)	*h* = −9→5
*T*_min_ = 0.629, *T*_max_ = 1	*k* = −16→15
7710 measured reflections	*l* = −15→16

### Refinement

**Table:**

Refinement on *F*^2^	Primary atom site location: structure-invariant direct methods
Least-squares matrix: full	Secondary atom site location: difference Fourier map
*R*[*F*^2^ > 2σ(*F*^2^)] = 0.040	Hydrogen site location: inferred from neighbouring sites
*wR*(*F*^2^) = 0.088	H-atom parameters constrained
*S* = 0.98	*w* = 1/[σ^2^(*F*_o_^2^) + (0.0429*P*)^2^] where *P* = (*F*_o_^2^ + 2*F*_c_^2^)/3
4651 reflections	(Δ/σ)_max_ < 0.001
305 parameters	Δρ_max_ = 1.74 e Å^−^^3^
0 restraints	Δρ_min_ = −0.93 e Å^−^^3^

### Special details

**Table:**

Geometry. All s.u.'s (except the s.u. in the dihedral angle between two l.s. planes) are estimated using the full covariance matrix. The cell s.u.'s are taken into account individually in the estimation of s.u.'s in distances, angles and torsion angles; correlations between s.u.'s in cell parameters are only used when they are defined by crystal symmetry. An approximate (isotropic) treatment of cell s.u.'s is used for estimating s.u.'s involving l.s. planes.
Refinement. Refinement of *F*^2^ against ALL reflections. The weighted *R*-factor *wR* and goodness of fit *S* are based on *F*^2^, conventional *R*-factors *R* are based on *F*, with *F* set to zero for negative *F*^2^. The threshold expression of *F*^2^ > 2σ(*F*^2^) is used only for calculating *R*-factors(gt) *etc*. and is not relevant to the choice of reflections for refinement. *R*-factors based on *F*^2^ are statistically about twice as large as those based on *F*, and *R*- factors based on ALL data will be even larger.

### Fractional atomic coordinates and isotropic or equivalent isotropic displacement parameters (Å^2^)

**Table:**

	*x*	*y*	*z*	*U*_iso_*/*U*_eq_	
Au	0.13463 (4)	0.49437 (2)	0.20439 (2)	0.03125 (11)	
Fe	0.5000	0.5000	0.0000	0.0270 (3)	
S1	0.1355 (3)	0.63009 (17)	0.35458 (14)	0.0434 (5)	
P1	0.1284 (2)	0.35677 (14)	0.05834 (13)	0.0266 (4)	
O1	0.4456 (6)	0.6118 (4)	0.4008 (3)	0.0351 (11)	
N1	0.3945 (9)	0.7445 (5)	0.5308 (5)	0.0457 (17)	
C1	0.3418 (10)	0.6707 (6)	0.4401 (6)	0.0382 (18)	
C2	0.2892 (11)	0.8073 (7)	0.5704 (6)	0.046 (2)	
C3	0.2959 (12)	0.8999 (7)	0.5460 (7)	0.057 (2)	
H3	0.3647	0.9185	0.4993	0.069*	
C4	0.2016 (12)	0.9663 (7)	0.5898 (7)	0.059 (2)	
H4	0.2036	1.0270	0.5689	0.071*	
C5	0.1061 (12)	0.9464 (8)	0.6622 (6)	0.056 (2)	
C6	0.1049 (13)	0.8551 (9)	0.6871 (6)	0.062 (3)	
H6	0.0406	0.8381	0.7363	0.075*	
C7	0.1941 (12)	0.7875 (8)	0.6427 (7)	0.056 (2)	
H7	0.1897	0.7259	0.6625	0.068*	
C8	0.0014 (15)	1.0160 (9)	0.7055 (8)	0.087 (4)	
H8A	−0.1098	0.9924	0.6548	0.130*	
H8B	0.0657	1.0926	0.7178	0.130*	
H8C	−0.0201	1.0084	0.7723	0.130*	
C9	0.6271 (10)	0.6486 (7)	0.4650 (6)	0.046 (2)	
H9	0.6256	0.6677	0.5414	0.055*	
C10	0.6848 (11)	0.5484 (7)	0.4295 (6)	0.047 (2)	
H10A	0.6075	0.4880	0.4441	0.071*	
H10B	0.8042	0.5666	0.4682	0.071*	
H10C	0.6794	0.5267	0.3536	0.071*	
C11	0.7391 (11)	0.7471 (7)	0.4477 (7)	0.060 (2)	
H11A	0.6955	0.8079	0.4726	0.091*	
H11B	0.7357	0.7298	0.3723	0.091*	
H11C	0.8590	0.7680	0.4870	0.091*	
C12	0.2458 (8)	0.4006 (5)	−0.0322 (5)	0.0263 (15)	
C13	0.2562 (9)	0.5014 (6)	−0.0501 (5)	0.0329 (16)	
H13	0.2079	0.5535	−0.0145	0.039*	
C14	0.3500 (10)	0.5101 (7)	−0.1288 (6)	0.044 (2)	
H14	0.3747	0.5680	−0.1558	0.053*	
C15	0.4004 (10)	0.4153 (7)	−0.1600 (5)	0.045 (2)	
H15	0.4655	0.3996	−0.2116	0.054*	
C16	0.3375 (9)	0.3480 (6)	−0.1013 (5)	0.0354 (17)	
H16	0.3534	0.2804	−0.1070	0.043*	
C17	−0.0903 (8)	0.2718 (5)	−0.0236 (5)	0.0267 (15)	
C18	−0.2168 (10)	0.2284 (6)	0.0247 (6)	0.0423 (19)	
H18	−0.1893	0.2431	0.0985	0.051*	
C19	−0.3841 (11)	0.1631 (7)	−0.0356 (8)	0.058 (2)	
H19	−0.4695	0.1317	−0.0030	0.070*	
C20	−0.4260 (11)	0.1439 (7)	−0.1435 (8)	0.057 (2)	
H20	−0.5399	0.0997	−0.1843	0.068*	
C21	−0.3025 (11)	0.1890 (6)	−0.1906 (7)	0.049 (2)	
H21	−0.3323	0.1767	−0.2638	0.059*	
C22	−0.1347 (10)	0.2522 (6)	−0.1327 (6)	0.0384 (18)	
H22	−0.0499	0.2822	−0.1663	0.046*	
C23	0.2152 (8)	0.2599 (5)	0.0958 (5)	0.0294 (15)	
C24	0.3196 (10)	0.2918 (7)	0.1944 (6)	0.0423 (19)	
H24	0.3507	0.3648	0.2416	0.051*	
C25	0.3806 (12)	0.2174 (8)	0.2258 (7)	0.059 (2)	
H25	0.4512	0.2398	0.2946	0.070*	
C26	0.3391 (12)	0.1131 (8)	0.1582 (7)	0.056 (2)	
H26	0.3840	0.0639	0.1797	0.067*	
C27	0.2318 (10)	0.0772 (6)	0.0580 (7)	0.048 (2)	
H27	0.2006	0.0036	0.0119	0.057*	
C28	0.1709 (9)	0.1516 (6)	0.0263 (6)	0.0400 (18)	
H28	0.0993	0.1289	−0.0422	0.048*	

### Atomic displacement parameters (Å^2^)

**Table:**

	*U*^11^	*U*^22^	*U*^33^	*U*^12^	*U*^13^	*U*^23^
Au	0.02798 (16)	0.03521 (17)	0.02566 (15)	0.00940 (12)	0.00453 (10)	0.00587 (11)
Fe	0.0229 (7)	0.0322 (8)	0.0227 (6)	0.0055 (6)	0.0028 (5)	0.0098 (6)
S1	0.0352 (11)	0.0527 (12)	0.0320 (9)	0.0184 (10)	0.0026 (8)	−0.0004 (9)
P1	0.0224 (9)	0.0272 (9)	0.0276 (9)	0.0064 (8)	0.0042 (7)	0.0086 (8)
O1	0.028 (3)	0.042 (3)	0.029 (2)	0.014 (2)	0.000 (2)	0.005 (2)
N1	0.046 (4)	0.048 (4)	0.033 (3)	0.020 (3)	−0.002 (3)	0.003 (3)
C1	0.040 (4)	0.035 (4)	0.033 (4)	0.003 (4)	0.003 (3)	0.015 (4)
C2	0.048 (5)	0.054 (5)	0.025 (4)	0.019 (4)	0.000 (4)	0.003 (4)
C3	0.064 (6)	0.051 (5)	0.056 (5)	0.016 (5)	0.028 (5)	0.016 (5)
C4	0.065 (6)	0.040 (5)	0.063 (6)	0.016 (5)	0.013 (5)	0.008 (4)
C5	0.054 (6)	0.068 (6)	0.027 (4)	0.018 (5)	−0.002 (4)	−0.005 (4)
C6	0.063 (6)	0.092 (8)	0.034 (4)	0.027 (6)	0.015 (4)	0.023 (5)
C7	0.053 (5)	0.075 (6)	0.050 (5)	0.032 (5)	0.005 (4)	0.028 (5)
C8	0.086 (8)	0.099 (8)	0.053 (6)	0.048 (7)	0.003 (6)	−0.015 (6)
C9	0.036 (4)	0.058 (5)	0.031 (4)	0.008 (4)	−0.002 (3)	0.011 (4)
C10	0.044 (5)	0.070 (6)	0.034 (4)	0.027 (4)	0.007 (4)	0.021 (4)
C11	0.043 (5)	0.051 (5)	0.064 (6)	−0.001 (4)	−0.001 (4)	0.009 (5)
C12	0.023 (3)	0.025 (3)	0.027 (3)	0.006 (3)	0.001 (3)	0.007 (3)
C13	0.025 (4)	0.037 (4)	0.035 (4)	0.010 (3)	−0.002 (3)	0.016 (3)
C14	0.043 (5)	0.055 (5)	0.032 (4)	0.007 (4)	−0.002 (4)	0.023 (4)
C15	0.037 (4)	0.064 (6)	0.018 (3)	0.002 (4)	0.005 (3)	0.007 (4)
C16	0.032 (4)	0.037 (4)	0.030 (4)	0.009 (3)	0.005 (3)	0.006 (3)
C17	0.026 (4)	0.016 (3)	0.037 (4)	0.007 (3)	0.003 (3)	0.009 (3)
C18	0.036 (4)	0.044 (5)	0.053 (5)	0.014 (4)	0.019 (4)	0.022 (4)
C19	0.036 (5)	0.044 (5)	0.104 (8)	0.011 (4)	0.029 (5)	0.035 (5)
C20	0.025 (4)	0.042 (5)	0.088 (7)	0.006 (4)	−0.009 (5)	0.016 (5)
C21	0.036 (5)	0.045 (5)	0.049 (5)	0.009 (4)	−0.011 (4)	0.006 (4)
C22	0.036 (4)	0.038 (4)	0.037 (4)	0.012 (4)	0.006 (3)	0.009 (3)
C23	0.018 (3)	0.028 (4)	0.035 (4)	−0.002 (3)	0.007 (3)	0.011 (3)
C24	0.041 (5)	0.054 (5)	0.036 (4)	0.021 (4)	0.007 (3)	0.019 (4)
C25	0.072 (6)	0.087 (7)	0.041 (5)	0.051 (6)	0.015 (4)	0.032 (5)
C26	0.066 (6)	0.070 (6)	0.072 (6)	0.045 (5)	0.037 (5)	0.052 (6)
C27	0.040 (5)	0.031 (4)	0.074 (6)	0.004 (4)	0.019 (4)	0.026 (4)
C28	0.025 (4)	0.037 (4)	0.052 (5)	0.004 (3)	0.003 (3)	0.017 (4)

### Geometric parameters (Å, °)

**Table:**

Au—S1	2.2883 (19)	C10—H10A	0.9700
Au—P1	2.2520 (17)	C10—H10B	0.9700
Fe—C13^i^	2.025 (6)	C10—H10C	0.9700
Fe—C13	2.025 (6)	C11—H11A	0.9700
Fe—C12	2.036 (6)	C11—H11B	0.9700
Fe—C12^i^	2.036 (6)	C11—H11C	0.9700
Fe—C16	2.039 (7)	C12—C16	1.420 (9)
Fe—C16^i^	2.039 (7)	C12—C13	1.433 (9)
Fe—C15	2.046 (7)	C13—C14	1.403 (10)
Fe—C15^i^	2.046 (7)	C13—H13	0.9400
Fe—C14^i^	2.059 (7)	C14—C15	1.416 (11)
Fe—C14	2.059 (7)	C14—H14	0.9400
S1—C1	1.753 (7)	C15—C16	1.411 (10)
P1—C12	1.784 (7)	C15—H15	0.9400
P1—C23	1.814 (7)	C16—H16	0.9400
P1—C17	1.818 (6)	C17—C18	1.377 (10)
O1—C1	1.370 (9)	C17—C22	1.396 (9)
O1—C9	1.479 (8)	C18—C19	1.382 (11)
N1—C1	1.253 (9)	C18—H18	0.9400
N1—C2	1.415 (10)	C19—C20	1.381 (13)
C2—C7	1.365 (12)	C19—H19	0.9400
C2—C3	1.373 (11)	C20—C21	1.358 (12)
C3—C4	1.390 (12)	C20—H20	0.9400
C3—H3	0.9400	C21—C22	1.371 (10)
C4—C5	1.369 (12)	C21—H21	0.9400
C4—H4	0.9400	C22—H22	0.9400
C5—C6	1.369 (13)	C23—C24	1.358 (9)
C5—C8	1.485 (12)	C23—C28	1.393 (10)
C6—C7	1.372 (12)	C24—C25	1.386 (10)
C6—H6	0.9400	C24—H24	0.9400
C7—H7	0.9400	C25—C26	1.346 (12)
C8—H8A	0.9700	C25—H25	0.9400
C8—H8B	0.9700	C26—C27	1.378 (12)
C8—H8C	0.9700	C26—H26	0.9400
C9—C11	1.488 (11)	C27—C28	1.387 (10)
C9—C10	1.519 (10)	C27—H27	0.9400
C9—H9	0.9900	C28—H28	0.9400
			
P1—Au—S1	178.17 (8)	O1—C9—C10	104.7 (6)
C13^i^—Fe—C13	180.0	C11—C9—C10	113.9 (7)
C13^i^—Fe—C12	138.7 (3)	O1—C9—H9	109.3
C13—Fe—C12	41.3 (3)	C11—C9—H9	109.3
C13^i^—Fe—C12^i^	41.3 (3)	C10—C9—H9	109.3
C13—Fe—C12^i^	138.7 (3)	C9—C10—H10A	109.5
C12—Fe—C12^i^	180.0	C9—C10—H10B	109.5
C13^i^—Fe—C16	111.4 (3)	H10A—C10—H10B	109.5
C13—Fe—C16	68.6 (3)	C9—C10—H10C	109.5
C12—Fe—C16	40.8 (3)	H10A—C10—H10C	109.5
C12^i^—Fe—C16	139.2 (3)	H10B—C10—H10C	109.5
C13^i^—Fe—C16^i^	68.6 (3)	C9—C11—H11A	109.5
C13—Fe—C16^i^	111.4 (3)	C9—C11—H11B	109.5
C12—Fe—C16^i^	139.2 (3)	H11A—C11—H11B	109.5
C12^i^—Fe—C16^i^	40.8 (3)	C9—C11—H11C	109.5
C16—Fe—C16^i^	180.0 (4)	H11A—C11—H11C	109.5
C13^i^—Fe—C15	112.4 (3)	H11B—C11—H11C	109.5
C13—Fe—C15	67.6 (3)	C16—C12—C13	106.7 (6)
C12—Fe—C15	68.2 (3)	C16—C12—P1	130.5 (5)
C12^i^—Fe—C15	111.8 (3)	C13—C12—P1	122.8 (5)
C16—Fe—C15	40.4 (3)	C16—C12—Fe	69.7 (4)
C16^i^—Fe—C15	139.6 (3)	C13—C12—Fe	68.9 (4)
C13^i^—Fe—C15^i^	67.6 (3)	P1—C12—Fe	127.9 (3)
C13—Fe—C15^i^	112.4 (3)	C14—C13—C12	109.3 (7)
C12—Fe—C15^i^	111.8 (3)	C14—C13—Fe	71.2 (4)
C12^i^—Fe—C15^i^	68.2 (3)	C12—C13—Fe	69.7 (4)
C16—Fe—C15^i^	139.6 (3)	C14—C13—H13	125.4
C16^i^—Fe—C15^i^	40.4 (3)	C12—C13—H13	125.4
C15—Fe—C15^i^	180.0 (2)	Fe—C13—H13	125.3
C13^i^—Fe—C14^i^	40.2 (3)	C13—C14—C15	107.0 (7)
C13—Fe—C14^i^	139.8 (3)	C13—C14—Fe	68.6 (4)
C12—Fe—C14^i^	111.2 (3)	C15—C14—Fe	69.3 (4)
C12^i^—Fe—C14^i^	68.8 (3)	C13—C14—H14	126.5
C16—Fe—C14^i^	111.6 (3)	C15—C14—H14	126.5
C16^i^—Fe—C14^i^	68.4 (3)	Fe—C14—H14	127.1
C15—Fe—C14^i^	139.6 (3)	C16—C15—C14	109.2 (7)
C15^i^—Fe—C14^i^	40.4 (3)	C16—C15—Fe	69.5 (4)
C13^i^—Fe—C14	139.8 (3)	C14—C15—Fe	70.3 (4)
C13—Fe—C14	40.2 (3)	C16—C15—H15	125.4
C12—Fe—C14	68.8 (3)	C14—C15—H15	125.4
C12^i^—Fe—C14	111.2 (3)	Fe—C15—H15	126.3
C16—Fe—C14	68.4 (3)	C15—C16—C12	107.9 (7)
C16^i^—Fe—C14	111.6 (3)	C15—C16—Fe	70.1 (4)
C15—Fe—C14	40.4 (3)	C12—C16—Fe	69.5 (4)
C15^i^—Fe—C14	139.6 (3)	C15—C16—H16	126.0
C14^i^—Fe—C14	180.000 (1)	C12—C16—H16	126.0
C1—S1—Au	105.7 (3)	Fe—C16—H16	126.0
C12—P1—C23	108.0 (3)	C18—C17—C22	119.3 (7)
C12—P1—C17	104.2 (3)	C18—C17—P1	118.6 (5)
C23—P1—C17	104.0 (3)	C22—C17—P1	122.1 (6)
C12—P1—Au	114.8 (2)	C17—C18—C19	119.9 (8)
C23—P1—Au	110.6 (2)	C17—C18—H18	120.0
C17—P1—Au	114.5 (2)	C19—C18—H18	120.0
C1—O1—C9	116.5 (5)	C20—C19—C18	120.1 (8)
C1—N1—C2	120.7 (7)	C20—C19—H19	120.0
N1—C1—O1	120.6 (7)	C18—C19—H19	120.0
N1—C1—S1	125.4 (6)	C21—C20—C19	120.0 (8)
O1—C1—S1	114.0 (5)	C21—C20—H20	120.0
C7—C2—C3	117.1 (8)	C19—C20—H20	120.0
C7—C2—N1	122.6 (8)	C20—C21—C22	120.8 (8)
C3—C2—N1	119.9 (8)	C20—C21—H21	119.6
C2—C3—C4	120.5 (9)	C22—C21—H21	119.6
C2—C3—H3	119.7	C21—C22—C17	119.8 (8)
C4—C3—H3	119.7	C21—C22—H22	120.1
C5—C4—C3	122.4 (9)	C17—C22—H22	120.1
C5—C4—H4	118.8	C24—C23—C28	119.2 (7)
C3—C4—H4	118.8	C24—C23—P1	119.8 (5)
C6—C5—C4	115.9 (9)	C28—C23—P1	121.0 (5)
C6—C5—C8	122.3 (10)	C23—C24—C25	120.4 (8)
C4—C5—C8	121.7 (10)	C23—C24—H24	119.8
C5—C6—C7	122.3 (9)	C25—C24—H24	119.8
C5—C6—H6	118.8	C26—C25—C24	120.2 (8)
C7—C6—H6	118.8	C26—C25—H25	119.9
C2—C7—C6	121.7 (9)	C24—C25—H25	119.9
C2—C7—H7	119.1	C25—C26—C27	121.1 (7)
C6—C7—H7	119.1	C25—C26—H26	119.5
C5—C8—H8A	109.5	C27—C26—H26	119.5
C5—C8—H8B	109.5	C26—C27—C28	118.7 (8)
H8A—C8—H8B	109.5	C26—C27—H27	120.6
C5—C8—H8C	109.5	C28—C27—H27	120.6
H8A—C8—H8C	109.5	C27—C28—C23	120.3 (7)
H8B—C8—H8C	109.5	C27—C28—H28	119.9
O1—C9—C11	110.2 (6)	C23—C28—H28	119.9
			
C2—N1—C1—O1	178.4 (7)	C16—Fe—C14—C13	81.9 (5)
C2—N1—C1—S1	−3.5 (11)	C16^i^—Fe—C14—C13	−98.1 (5)
C9—O1—C1—N1	−6.9 (10)	C15—Fe—C14—C13	118.9 (6)
C9—O1—C1—S1	174.8 (5)	C15^i^—Fe—C14—C13	−61.1 (6)
Au—S1—C1—N1	179.4 (6)	C13^i^—Fe—C14—C15	61.1 (6)
Au—S1—C1—O1	−2.4 (6)	C13—Fe—C14—C15	−118.9 (6)
C1—N1—C2—C7	101.8 (9)	C12—Fe—C14—C15	−80.9 (5)
C1—N1—C2—C3	−85.8 (10)	C12^i^—Fe—C14—C15	99.1 (5)
C7—C2—C3—C4	−3.1 (13)	C16—Fe—C14—C15	−37.0 (4)
N1—C2—C3—C4	−175.9 (7)	C16^i^—Fe—C14—C15	143.0 (4)
C2—C3—C4—C5	3.3 (14)	C15^i^—Fe—C14—C15	180.0
C3—C4—C5—C6	−1.7 (13)	C13—C14—C15—C16	0.3 (8)
C3—C4—C5—C8	−178.0 (9)	Fe—C14—C15—C16	58.8 (5)
C4—C5—C6—C7	0.2 (13)	C13—C14—C15—Fe	−58.5 (5)
C8—C5—C6—C7	176.4 (8)	C13^i^—Fe—C15—C16	97.3 (5)
C3—C2—C7—C6	1.7 (12)	C13—Fe—C15—C16	−82.7 (5)
N1—C2—C7—C6	174.3 (8)	C12—Fe—C15—C16	−38.0 (4)
C5—C6—C7—C2	−0.3 (14)	C12^i^—Fe—C15—C16	142.0 (4)
C1—O1—C9—C11	−78.4 (8)	C14^i^—Fe—C15—C16	59.6 (6)
C1—O1—C9—C10	158.6 (6)	C14—Fe—C15—C16	−120.4 (6)
C23—P1—C12—C16	24.5 (7)	C13^i^—Fe—C15—C14	−142.3 (4)
C17—P1—C12—C16	−85.7 (6)	C13—Fe—C15—C14	37.7 (4)
Au—P1—C12—C16	148.3 (5)	C12—Fe—C15—C14	82.4 (5)
C23—P1—C12—C13	−158.2 (5)	C12^i^—Fe—C15—C14	−97.6 (5)
C17—P1—C12—C13	91.7 (5)	C16—Fe—C15—C14	120.4 (6)
Au—P1—C12—C13	−34.4 (6)	C16^i^—Fe—C15—C14	−59.6 (6)
C23—P1—C12—Fe	−70.5 (5)	C14—C15—C16—C12	0.1 (8)
C17—P1—C12—Fe	179.3 (4)	Fe—C15—C16—C12	59.4 (5)
Au—P1—C12—Fe	53.3 (4)	C14—C15—C16—Fe	−59.3 (5)
C13^i^—Fe—C12—C16	−62.0 (6)	C13—C12—C16—C15	−0.5 (7)
C13—Fe—C12—C16	118.0 (6)	P1—C12—C16—C15	177.2 (5)
C16^i^—Fe—C12—C16	180.0	Fe—C12—C16—C15	−59.8 (5)
C15—Fe—C12—C16	37.6 (4)	C13—C12—C16—Fe	59.3 (4)
C15^i^—Fe—C12—C16	−142.4 (4)	P1—C12—C16—Fe	−123.0 (6)
C14^i^—Fe—C12—C16	−98.9 (4)	C13^i^—Fe—C16—C15	−99.8 (5)
C14—Fe—C12—C16	81.1 (4)	C13—Fe—C16—C15	80.2 (5)
C13^i^—Fe—C12—C13	180.0	C12—Fe—C16—C15	119.0 (6)
C16—Fe—C12—C13	−118.0 (6)	C12^i^—Fe—C16—C15	−61.0 (6)
C16^i^—Fe—C12—C13	62.0 (6)	C14^i^—Fe—C16—C15	−143.1 (5)
C15—Fe—C12—C13	−80.4 (4)	C14—Fe—C16—C15	36.9 (5)
C15^i^—Fe—C12—C13	99.6 (4)	C13^i^—Fe—C16—C12	141.2 (4)
C14^i^—Fe—C12—C13	143.1 (4)	C13—Fe—C16—C12	−38.8 (4)
C14—Fe—C12—C13	−36.9 (4)	C12^i^—Fe—C16—C12	180.0
C13^i^—Fe—C12—P1	64.2 (6)	C15—Fe—C16—C12	−119.0 (6)
C13—Fe—C12—P1	−115.8 (6)	C15^i^—Fe—C16—C12	61.0 (6)
C16—Fe—C12—P1	126.1 (6)	C14^i^—Fe—C16—C12	97.9 (4)
C16^i^—Fe—C12—P1	−53.9 (6)	C14—Fe—C16—C12	−82.1 (4)
C15—Fe—C12—P1	163.8 (5)	C12—P1—C17—C18	−177.4 (5)
C15^i^—Fe—C12—P1	−16.2 (5)	C23—P1—C17—C18	69.6 (6)
C14^i^—Fe—C12—P1	27.3 (5)	Au—P1—C17—C18	−51.2 (6)
C14—Fe—C12—P1	−152.7 (5)	C12—P1—C17—C22	0.7 (6)
C16—C12—C13—C14	0.7 (7)	C23—P1—C17—C22	−112.3 (6)
P1—C12—C13—C14	−177.2 (5)	Au—P1—C17—C22	126.9 (5)
Fe—C12—C13—C14	60.5 (5)	C22—C17—C18—C19	2.1 (10)
C16—C12—C13—Fe	−59.8 (4)	P1—C17—C18—C19	−179.7 (6)
P1—C12—C13—Fe	122.3 (5)	C17—C18—C19—C20	−1.8 (12)
C12—Fe—C13—C14	−119.8 (6)	C18—C19—C20—C21	0.2 (13)
C12^i^—Fe—C13—C14	60.2 (6)	C19—C20—C21—C22	1.1 (12)
C16—Fe—C13—C14	−81.5 (5)	C20—C21—C22—C17	−0.8 (12)
C16^i^—Fe—C13—C14	98.5 (5)	C18—C17—C22—C21	−0.8 (10)
C15—Fe—C13—C14	−37.8 (5)	P1—C17—C22—C21	−178.9 (6)
C15^i^—Fe—C13—C14	142.2 (5)	C12—P1—C23—C24	106.1 (6)
C14^i^—Fe—C13—C14	180.0	C17—P1—C23—C24	−143.6 (6)
C12^i^—Fe—C13—C12	180.0	Au—P1—C23—C24	−20.2 (6)
C16—Fe—C13—C12	38.3 (4)	C12—P1—C23—C28	−76.4 (6)
C16^i^—Fe—C13—C12	−141.7 (4)	C17—P1—C23—C28	33.9 (7)
C15—Fe—C13—C12	82.0 (4)	Au—P1—C23—C28	157.3 (5)
C15^i^—Fe—C13—C12	−98.0 (4)	C28—C23—C24—C25	−0.3 (12)
C14^i^—Fe—C13—C12	−60.2 (6)	P1—C23—C24—C25	177.2 (6)
C14—Fe—C13—C12	119.8 (6)	C23—C24—C25—C26	1.0 (13)
C12—C13—C14—C15	−0.6 (7)	C24—C25—C26—C27	−1.8 (14)
Fe—C13—C14—C15	59.0 (5)	C25—C26—C27—C28	1.9 (13)
C12—C13—C14—Fe	−59.6 (4)	C26—C27—C28—C23	−1.2 (12)
C13^i^—Fe—C14—C13	180.0	C24—C23—C28—C27	0.4 (11)
C12—Fe—C14—C13	37.9 (4)	P1—C23—C28—C27	−177.0 (6)
C12^i^—Fe—C14—C13	−142.1 (4)		

Symmetry codes: (i) −*x*+1, −*y*+1, −*z*.

### Hydrogen-bond geometry (Å, °)

**Table:**

*D*—H···*A*	*D*—H	H···*A*	*D*···*A*	*D*—H···*A*
C25—H25···N1^ii^	0.94	2.45	3.370 (11)	167

Symmetry codes: (ii) −*x*+1, −*y*+1, −*z*+1.
